# Guillain‐Barré Syndrome in Late Pregnancy Following Respiratory Syncytial Virus Infection: A Diagnostic Dilemma

**DOI:** 10.1002/ccr3.73817

**Published:** 2026-10-28

**Authors:** Rhiana Pereira‐Lindley, Mesharck Gariba, Darshan Lal

**Affiliations:** ^1^ Bedford Hospital Bedford UK; ^2^ School of Medicine and Population Health University of Sheffield Sheffield UK

**Keywords:** electrodiagnosis, Guillain–Barré syndrome, nerve conduction studies, neuromuscular disease, pregnancy complications, respiratory syncytial virus infections

## Abstract

The diagnosis of Guillain–Barré syndrome (GBS) relies on evolving clinical features, supported by cerebrospinal fluid (CSF), laboratory investigations, and electrodiagnostic studies. Early investigations may be nondiagnostic and should not delay treatment when progressive neurological deterioration raises clinical suspicion of GBS. Timely treatment is crucial in optimizing maternal and fetal outcomes.

## 1 Introduction

A woman in her late 30s at 34 weeks' gestation presented with progressive lower limb weakness, patchy sensory loss, and incontinence following a recent respiratory syncytial virus (RSV) infection. Initial differential diagnoses included cauda equina syndrome (CES), transverse myelitis, and GBS. Magnetic resonance imaging (MRI) and nerve conduction studies (NCS) were nondiagnostic, contributing to diagnostic uncertainty. Lumbar puncture demonstrated albuminocytological dissociation, supporting a working diagnosis of GBS [1]. Based on the clinical presentation, this patient met Brighton Level 2 diagnostic criteria for GBS.

GBS in pregnancy is rare and may be difficult to recognize, as its early manifestations can overlap with physiological changes of pregnancy. Despite increased maternal and perinatal risk, favorable fetal outcomes have been reported with timely management [2, 3]. Although CSF and electrodiagnostic studies provide supporting evidence, early studies may be normal during the first weeks of GBS. This reinforces the need to interpret findings in the context of evolving clinical features.

This case highlights the importance of individualized clinical decision‐making, multidisciplinary teamwork, and early initiation of treatment when progressive neurological deterioration suggests GBS despite inconclusive early investigations.

## 2 Case History

A P3, 34 + 3‐week‐pregnant woman in her late 30s with a background of hypothyroidism on levothyroxine was referred by her general practitioner (GP) with suspected CES due to worsening bilateral leg numbness and urinary and fecal incontinence. Over the following days, the sensory disturbance ascended to involve both hands, and she was unable to perceive fetal movements. She subsequently developed bulbar involvement with dysphagia and a left lower motor neuron facial nerve (CN VII) palsy (Table 1).

She reported a preceding history of sore throat and altered taste sensation. Systemic examination revealed an inflamed pharynx and enlarged tonsils, and nasopharyngeal testing confirmed RSV infection. She had also received a routine maternal RSV vaccination 11 days before the onset of her neurological symptoms.

Examination revealed mild hypotonia, more pronounced in the lower than upper extremities, with power of 4−/5 in the lower limbs and 4+/5 in the upper limbs, along with mild bilateral handgrip weakness. Deep tendon reflexes were absent throughout, and anal tone was absent. A T6 sensory level was documented during the early admission; however, later examination demonstrated patchy and inconsistent sensory loss in the lower limbs.

### 2.1 Timeline

**TABLE 1:** Chronological timeline of events.

Day 0	Routine maternal RSV vaccination
Day 1–7 (Exact date unspecified)	Sore throat, altered taste
Day 8	Neurological symptom onset: Bilateral leg numbness and urinary and fecal incontinence
Day 11	First presentation following GP referral MRI lumbosacral spine performed RSV positive Discharged with planned follow‐up
Day 15	Second presentation and hospital admission Sensory disturbance had ascended to hands, unable to perceive fetal movements
Day 19	MRI whole spine performed
Day 20	New dysphagia and left LMN CN VII palsy Commenced prednisolone for suspected Bell's palsy
Day 24	MRI head
Day 25	Approximate nadir of neurological symptoms: unable to stand unsupported LP performed
Day 27	NCS/EMG performed
Day 32	IVIG commenced by neurology due to high clinical suspicion of GBS
Day 41	Induction of labor
Day 48	Discharge

### 2.2 Investigations

Written consent was obtained for the publication of this report and for the sharing of relevant clinical details and investigations.

MRI of the brain revealed no parenchymal abnormalities. MRI of the lumbosacral and whole spine showed no disc herniation, nerve root compression, or neural compromise. The MRI reports did not specify the use of intravenous contrast.

Within 2 weeks of admission, nerve conduction studies (NCS) showed evidence of left CN VII axonal impairment, with a reduced left nasalis compound muscle action potential (CMAP), consistent with observed CN VII palsy (Table 2). Severe bilateral carpal tunnel lesions (*R* > L) were also noted (Table 3). Initial motor conduction studies did not demonstrate electrophysiological evidence of demyelinating or axonal polyneuropathy (Table 2), and F‐wave latencies were also nondiagnostic at this stage (Table 4). Electromyography findings were unremarkable, with no evidence of denervation (Table 5). Blink reflex studies and anti‐ganglioside antibodies were not obtained in this case.

**TABLE 2:** Motor nerve conduction studies show evidence of left CN VII axonal impairment with a reduced left nasalis CMAP.

Motor nerves	Stim–rec	Onset lat [ms]	Amp O–P [mV]	Duration [ms]	Area [mV*ms]	Stim intensity [mA]	Stim duration [μs]	Dist [mm]	CV [m/s]
Median APB–Right	Wrist–APB	7.7	3.2	24.6	19.5	30.0	100	—	—
Median APB–Left	Wrist–APB	5.9	3.6	24.2	24.4	30.0	100	—	—
Ulnar ADM–Right	Wrist—ADM	3.3	5.8	20.5	36.4	26.0	100		
	B. Elbow–Wrist	7.4	3.4	20.2	21.3	30.0	100	200	49
	A. Elbow–B. Elbow	9.0	3.1	24.8	21.3	18.0	100	100	59
Ulnar ADM–Left	Wrist–ADM	3.0	6.7	21.4	43.5	30.0	100		
	B. Elbow–Wrist	7.2	4.6	20.1	27.7	36.0	100	200	48
	A. Elbow–B. Elbow	8.9	4.8	20.4	28.4	44.0	250	100	58
Facial–Right	Tragus–Nasalis	3.6	0.5	24.1	3.0	32.0	100	—	—
Facial–Left	Tragus–Nasalis	4.6	0.2	11.3	0.6	26.0	100	—	—
Peroneal EDB–Right	Ankle–EDB	7.4	1.7	20.8	11.0	50.0	500	—	—
	B. Fib Neck—Ankle	13.8	1.2	23.7	8.2	50.0	400	300	47
	A. Fib Neck–B. Fib Neck	15.4	1.0	28.2	7.2	42.0	300	90	59
Peroneal EDB–Left	Ankle–EDB	7.1	1.4	25.7	11.2	65.0	300	—	—
Tibial AH–Right	Ankle–AH	6.6	5.8	20.3	43.4	70.0	500	—	—
Tibial AH–Left	Ankle–AH	6.6	5.0	23.7	23.0	60.0	750	—	—

Abbreviations: ADM, abductor digiti minimi; AH, abductor hallucis; Amp, amplitude; APB, abductor pollicis brevis; CMAP, compound muscle action potential; CV, conduction velocity; Dist, distance; EDB, extensor digitorum brevis; Fib, Fibular; lat, latency; O–P, onset to peak; rec, recording; Stim, stimulation.

**TABLE 3:** Sensory nerve conduction studies demonstrate severe bilateral carpal tunnel syndrome *R* > L.

Sensory nerves	Stim–rec	Onset Lat [ms]	Amp T–P [μV]	Duration [ms]	Stim intensity [mA]	Stim duration [μs]	Dist [mm]	CV [m/s]
Median–Right	F2–Wrist	NR	NR	NR	NR	NR	NR	NR
Median–Left	F2–Wrist	NR	NR	NR	NR	NR	NR	NR
Ulnar–Right	F5–Wrist	1.5	3.6	—	24.0	100	90	61
Ulnar Left	F5–Wrist	1.4	4.0	—	30.0	100	90	66
Radial–Right	Snuff Box–forearm	1.6	26.5	—	22.0	100	90	56
Sural–Right	Lower leg–Lat Mall	1.7	22.8	—	19.0	100	90	54
Superficial Peroneal–Right	Lower leg–ankle	1.4	13.5	—	22.0	100	80	59
Superficial Peroneal–Left	Lower leg–ankle	1.2	15.8	—	42.0	100	—	—

Abbreviations: Amp, amplitude; CV, conduction velocity; Lat, latency; Lat mall, lateral malleolus; NR, no response; stim, stimulation; T–P, trough‐to‐peak.

**TABLE 4:** F‐wave nerves.

F‐wave nerves	M‐latency [ms]	M‐amplitude [mV]	Fmin [ms]	F–M [ms]
Median APB–Right	7.9	3.4	35.3	27.3
Median APB–Left	6.2	3.8	34.7	28.5
Ulnar ADM–Right	3.4	6.0	29.0	25.7
Ulnar ADM–Left	3.1	7.1	30.8	27.7
Peroneal EDB–Right	7.6	1.6	43.9	36.3
Peroneal EDB–Left	7.5	1.5	44.7	37.2
Tibial AH–Right	6.7	6.0	43.8	37.1
Tibial AH–Left	1.9	5.0	42.5	40.6

Abbreviations: ADM, abductor digiti minimi; AH, abductor hallucis; APB, abductor pollicis brevis; EDB, extensor digitorum brevis; F–M, F‐wave minus M‐wave latency; Fmin, minimum F‐wave latency.

**TABLE 5:** EMG scoring.

EMG scoring														
Insert‐activity	Spontaneous activity						Voluntary activity							Interpretation
	Fibril	PSW	Fascic.	Myot.	Myok.	CRD	Amp	Durat.	Polyph.	Stable	IP	Recruit.	Effort	
Tibialis anterior–Left														
0	0	0	0	0	0	0	N	N	N	N	Full	N	Good	Normal
Vastus medialis–Right														
0	0	0	0	0	0	0	N	N	N	N	Full	N	Good	Normal
Tibialis anterior–Right														
0	0	0	0	0	0	0	N	N	N	N	Full	N	Good	Normal

Abbreviations: Amp, amplitude; CRD, complex repetitive discharges; Durat., duration; EMG, electromyography; Fascic, fasciculations; fibril, fibrillation potentials; IP, interference pattern; Myok, myokymia; Myot, myotonia; Polyph., polyphasia; PS, positive sharp waves; Recruit, Recruitment.

Lumbar puncture was performed 2 weeks after neurological symptom onset. CSF analysis demonstrated albuminocytological dissociation, with an elevated CSF protein level (0.50 g/L; reference range 0–0.4 g/L) and a normal white cell count, findings supporting GBS [1].

Serial blood tests demonstrated marked transaminitis (peak ALT 813 U/L; reference range 0–32 U/L), with normal bilirubin. An extensive liver workup, including viral, autoimmune, and Wilson's disease screening, was unremarkable. Liver function gradually improved, and it was agreed that the transaminitis likely represented a transient viral‐related etiology.

### 2.3 Differential Diagnoses

Initial bilateral lower limb weakness, absent anal tone, and a T6 sensory level raised concern for CES, GBS, or transverse myelitis. MRI of the lumbosacral spine on admission showed no evidence of any structural abnormality or nerve root compression; both the cauda equina and conus medullaris were unremarkable. One week later, a whole‐spine MRI showed no neural compromise, disc disease, or radiological features suggestive of transverse myelitis. However, a normal early MRI cannot definitively exclude inflammatory myelopathy. CSF did not show a raised white cell count, and subsequent sensory examinations did not consistently demonstrate a T6 level, reducing the clinical suspicion of an inflammatory spinal pathology.

The atypical early T6 sensory level, sphincter dysfunction, inconsistent motor findings, and nondermatomal sensory loss were not typical of GBS and exacerbated diagnostic uncertainty. This prompted referral to psychiatric liaison services for consideration of functional neurological disorder (FND). However, serial neurological examination demonstrated progressive, objective neurological deficits including lower limb weakness, global hyporeflexia, autonomic dysfunction, and CN VII involvement. This was considered less suggestive of FND and supported an organic etiology. GBS was subsequently favored, with CSF findings providing additional support for this leading diagnosis.

The combination of albuminocytological dissociation, bilateral flaccid limb weakness, absent deep tendon reflexes, a monophasic illness course, and no alternative explanation for the weakness met Brighton Level 2 diagnostic certainty for GBS. The antecedent RSV infection was considered a potential trigger.

In the context of transaminitis, other possible differential diagnoses, such as hemolysis, elevated liver enzymes, and low platelet count (HELLP) syndrome and Wilson's disease were considered but excluded based on normal liver ultrasound, platelets, and copper studies.

### 2.4 Treatment

Following the development of CN VII palsy, oral prednisolone 60 mg with a proton pump inhibitor (PPI) was commenced for 7 days. In view of her progressive symptoms and increasing clinical suspicion of GBS, she was reviewed by a neurologist who commenced a 5‐day course of intravenous immunoglobulin (IVIG). Standard IVIG dosing is 2 g/kg spread over 5 days. In this case, the patient (57 kg ideal body weight) received a 20 g/day course, adjusted in accordance with local guidelines.

She was referred to the neurophysiotherapy team for rehabilitation, and to the speech and language therapy team, who assessed her swallowing and commenced a soft diet. She was reviewed regularly by obstetricians, with cardiotocography conducted throughout admission. Labour was induced at 38 weeks, resulting in delivery of a healthy infant.

## 3 Conclusion and Result

Following IVIG treatment, neurological improvement was observed. She was able to mobilize short distances and independently care for her infant. She was deemed medically fit for discharge 16 days after IVIG treatment was initiated, by which time she had experienced marked improvement in her symptoms. She was able to walk with minimal support and had resumed her regular diet. Her CN VII paresis resolved completely within approximately 1 week. She was scheduled for a neurology outpatient clinic follow‐up in 2 months.

## 4 Discussion

Guillain–Barré syndrome is a rare but serious immune‐mediated neuropathy affecting the peripheral nervous system. It is thought to arise through molecular mimicry, where antibodies directed at infectious agents cross‐react with peripheral nerve components, causing demyelination and axonal damage [4]. It classically presents with acute, flaccid ascending weakness, hyporeflexia, and paraesthesia, which may later progress to involve CN VII and bulbar dysfunction [1, 5]. It is estimated to occur in 1–2 per 100,000 annually, with more than 10% of patients experiencing ongoing severe disability and mortality rates reported around 5% [4, 5]. In pregnancy, GBS is uncommon, but most frequently presents during the third trimester.

This case report discusses several diagnostic pitfalls. Firstly, the interpretation of early symptoms may be impacted by pregnancy because they may initially be attributed to physiological gestational changes [6]. For example, bladder dysfunction in GBS more commonly presents as urinary retention due to autonomic neuropathy causing detrusor underactivity. Incontinence, as in this case, is less commonly reported. The potential overlap of pregnancy changes may have contributed to this presentation and obscured symptom interpretation. Similarly, reduced functional residual capacity in pregnancy can complicate respiratory assessment. Secondly, early MRI showed no compressive pathology, while early electrodiagnostic studies did not demonstrate features of GBS, increasing diagnostic uncertainty and prompting consideration of alternative differentials. Ultimately, the evolution of objective neurological findings, rather than a single supporting investigation, guided diagnosis and treatment. This reinforces that GBS remains a clinical diagnosis supported by investigations rather than defined by them [5].

The revised National Institute of Neurological Disorders and Stroke (NINDS) and the Brighton criteria are commonly used to guide diagnosis [7, 8]. Although early electrodiagnostic findings limited classification at the highest Brighton level, this patient's presentation fulfilled the criteria for a Brighton Level 2 diagnostic certainty. A raised CSF protein (> 0.4 g/L) with a normal white cell count in patients presenting with acute ascending paresis is suggestive of GBS [1]. A retrospective study by Hegen et al. [9] involving 97 patients with GBS in a local hospital emphasizes that true positives for CSF total protein and albumin quotient are most useful when interpreted in the context of early clinical suspicion. In this patient, the lumbar puncture was the main evidence supporting the decision to treat her for GBS. While this is a classical feature of GBS, it is important to acknowledge that it may be normal in the early disease stages [10].

Approximately two‐thirds of patients report symptoms of gastrointestinal or respiratory infection within the 6 weeks preceding the onset of GBS [11]. The common pathogens temporally associated with GBS in case–control studies include *Campylobacter jejuni* , Zika virus, Epstein–Barr virus, cytomegalovirus, hepatitis E virus, and *Mycoplasma pneumoniae* . This patient had received RSV vaccination 11 days before symptom onset and tested positive for RSV in the period between her vaccination and neurological symptoms. RSV infection can be a precipitant of GBS [12]. While temporal associations between RSV vaccination and GBS have been documented, we cannot infer a causal relationship in this case [13]. Given the established association between respiratory infections and GBS, RSV infection is considered the most likely precipitant.

Current European guidelines for the treatment of GBS recommend either IVIG, which acts through immunomodulation, or plasma exchange, which removes pathogenic proteins [14]. Both are efficacious; treatment is most effective when given as early as possible, particularly within 2 weeks of symptom onset [15]. In this case, diagnostic uncertainty persisted, and IVIG was initiated approximately 3 weeks after symptom onset, once progressive objective neurological deficits and supportive CSF findings increased suspicion of GBS. This reinforces the importance of initiating treatment promptly based on clinical suspicion and assessing alternative diagnoses rather than waiting for definitive confirmation.

### 4.1 Learning Points

- GBS in pregnancy is rare and may mimic physiological gestational symptoms
- Early negative investigations do not exclude GBS; diagnosis remains clinical, supported by investigations.
- Antecedent infections, including rarer triggers such as RSV, should be considered.
- Timely recognition and MDT management are crucial when clinical suspicion remains high despite early inconclusive investigations.

## 5 Patient Perspective

During my eighth month of pregnancy, I received a message from my GP and midwife regarding the RSV vaccination, which I received without any immediate concerns. Approximately 2 weeks later, I began to notice weakness in my legs. Simple activities such as climbing stairs became increasingly difficult, and I had to rely on support from railings. Initially, I attributed this to the advanced stage of pregnancy and increased body weight. However, my symptoms gradually worsened, prompting hospital attendance.

Many of my initial investigations were normal, which was both confusing and frustrating. During my hospital stay, I was reviewed by multiple teams, including medical, obstetric, and neurological specialists. I developed facial weakness, difficulty swallowing, and progressive limb weakness. What was particularly distressing was the rapid decline—from being able to walk with support to becoming bedbound within a short period. I recall a moment when the physiotherapy team asked me to stand, and my legs gave way completely. That experience was frightening and marked a turning point in my illness. I became entirely dependent on support for basic movements, which led to emotional distress, as each day brought new symptoms without a clear diagnosis.

It was only after repeated neurological assessments that I was diagnosed with Guillain–Barré syndrome, approximately 2 weeks after my admission. I was started on intravenous immunoglobulin (IVIG) treatment. I began to notice gradual changes—initially increased sensitivity and pain in my legs, followed by slight improvement in movement, such as the ability to bend my knees. Although the pain was significant, it was also a sign of recovery.

Throughout this period, I remained in the late stages of pregnancy. A growth scan later indicated reduced fetal growth, which added to further anxiety. In discussion with the obstetric team, a decision was made to proceed with the induction of labor. Despite my physical limitations, I was determined to give my best effort during delivery, and with support, I successfully delivered a healthy baby boy. This was a deeply emotional and positive outcome following a very challenging journey.

Reflecting on my hospital experience, I would like to highlight the important role of the multidisciplinary team. The medical team ensured thorough investigations and ongoing monitoring. The obstetric and midwifery teams, along with the nursing team, provided continuous support throughout pregnancy and delivery. The neurology team, particularly the consultant involved, played a crucial role in reaching the diagnosis and initiating treatment. I would especially like to acknowledge the physiotherapy and occupational therapy teams. Their encouragement, consistent input, and reassurance gave me hope during a very difficult time. Their belief that I would regain my strength played a significant role in my recovery journey.

Although the initial phase of uncertainty and normal investigations was distressing, I began to improve following treatment and delivery. Recovery has been gradual, and I continue to undergo rehabilitation. This experience has shown me how complex and unpredictable medical conditions can be, particularly in pregnancy, and how essential a coordinated, multidisciplinary approach is in managing such cases. Progressive neurological symptoms in pregnancy should not be attributed solely to physiological changes. Maternal deterioration and fetal concerns require balanced, timely obstetric decisions.

## Author Contributions

**Rhiana Pereira‐Lindley:** conceptualization, writing – original draft, writing – review and editing, project administration, data curation. **Mesharck Gariba:** writing – original draft, writing – review and editing, data curation. **Darshan Lal:** validation, supervision, writing – review and editing.

## Funding

The authors have nothing to report.

## Consent

Written informed consent was gained from this patient for publication of this case report including investigations and the patient perspective.

## Conflicts of Interest

The authors declare no conflicts of interest.

## Data Availability

Data sharing not applicable to this article as no datasets were generated or analysed during the current study.
